# Mapping QTLs for blight resistance and morpho-phenological traits in inter-species hybrid families of chestnut (*Castanea* spp.)

**DOI:** 10.3389/fpls.2024.1365951

**Published:** 2024-04-08

**Authors:** Shenghua Fan, Laura L. Georgi, Frederick V. Hebard, Tetyana Zhebentyayeva, Jiali Yu, Paul H. Sisco, Sara F. Fitzsimmons, Margaret E. Staton, Albert G. Abbott, C. Dana Nelson

**Affiliations:** ^1^ Forest Health Research and Education Center, Department of Horticulture, University of Kentucky, Lexington, KY, United States; ^2^ Forest Health Research and Education Center, U.S. Department of Agriculture (USDA) Forest Service, Southern Research Station, Lexington, KY, United States; ^3^ Virginia Chapter, The American Chestnut Foundation, Meadowview, VA, United States; ^4^ Forest Health Research and Education Center, Department of Forestry and Natural Resources, University of Kentucky, Lexington, KY, United States; ^5^ Synthetic and Systems Biology Innovation Hub, Department of Plant Pathology and Microbiology, Texas A&M University, College Station, TX, United States; ^6^ Carolinas Chapter, The American Chestnut Foundation, Asheville, NC, United States; ^7^ North Central Office, The American Chestnut Foundation, University Park, PA, United States; ^8^ Department of Entomology and Plant Pathology, University of Tennessee, Knoxville, TN, United States; ^9^ Southern Institute of Forest Genetics, U.S. Department of Agriculture (USDA) Forest Service, Southern Research Station, Saucier, MS, United States

**Keywords:** chestnut, Castanea, blight resistance, morphology, phenology, genetic architecture, GWAS, multiple QTL mapping

## Abstract

Chestnut blight (caused by *Cryphonectria parasitica*), together with *Phytophthora* root rot (caused by *Phytophthora cinnamomi*), has nearly extirpated American chestnut (*Castanea dentata*) from its native range. In contrast to the susceptibility of American chestnut, many Chinese chestnut (*C. mollissima*) genotypes are resistant to blight. In this research, we performed a series of genome-wide association studies for blight resistance originating from three unrelated Chinese chestnut trees (Mahogany, Nanking and M16) and a Quantitative Trait Locus (QTL) study on a Mahogany-derived inter-species F2 family. We evaluated trees for resistance to blight after artificial inoculation with two fungal strains and scored nine morpho-phenological traits that are the hallmarks of species differentiation between American and Chinese chestnuts. Results support a moderately complex genetic architecture for blight resistance, as 31 QTLs were found on 12 chromosomes across all studies. Additionally, although most morpho-phenological trait QTLs overlap or are adjacent to blight resistance QTLs, they tend to aggregate in a few genomic regions. Finally, comparison between QTL intervals for blight resistance and those previously published for *Phytophthora* root rot resistance, revealed five common disease resistance regions on chromosomes 1, 5, and 11. Our results suggest that it will be difficult, but still possible to eliminate Chinese chestnut alleles for the morpho-phenological traits while achieving relatively high blight resistance in a backcross hybrid tree. We see potential for a breeding scheme that utilizes marker-assisted selection early for relatively large effect QTLs followed by genome selection in later generations for smaller effect genomic regions.

## Introduction

Forest health is in decline in the US and around the world, driven by habitat loss and climate change, and invasive pests and pathogens. Despite the significant impacts of invasive pests and pathogens on forest tree species, relatively little has been done to improve forest trees’ genetic-based resistance. The ongoing saga of the near extirpation of the American chestnut (*Castanea dentata*) in North America and subsequent efforts to restore and sustain this formerly abundant species is a poignant example of the complexity of the issues surrounding genetic improvement strategies to alleviate pressure from invasive pests and pathogens. Thus, resistance breeding and genetically informed restoration efforts for the American chestnut provide a case study for investigating potential improvement of other forest tree species, as well as overall forest health and resilience.

American chestnut was at one time a foundational tree species throughout the oak-hickory forest types of the eastern United States (Russell, 1987). The species was a significant contributor to local economies, valued for both lumber and nut production (Buttrick, 1925; Cameron, 2002). With the invasion of the chestnut blight fungus *Cryphonectria parasitica* at the beginning of the 20^th^ century, most of the estimated billions of American chestnut trees were killed within a 40-year period (Anagnostakis, 1987; Dalgleish et al., 2016). At the same time, it became known that substantial levels of resistance to blight are present in Asian species of *Castanea*, including Chinese chestnut (*C. mollissima*), and Japanese chestnut (*C. crenata*) (Graves, 1950; Anagnostakis, 1992). Early genetic work on chestnut blight resistance suggested that resistance found in the Asian species was incompletely dominant and limited to two major loci (Clapper, 1952, 1954; Burnham et al., 1986). Given this and the cultural and ecological significance of American chestnut to the Appalachian Mountain region, the American Chestnut Foundation (TACF) initiated a backcross breeding strategy to introgress blight resistance from Chinese chestnut into American chestnut (Burnham, 1988).

In TACF’s breeding program, trees from backcross generations were primarily selected for blight resistance and secondarily for phenotypic traits that taxonomically distinguish the two chestnut species. Backcross breeding is most efficient when the target trait (i.e., resistance) is controlled by one or a small number of genes, thus it is important to understand the genetic architecture of resistance. As the number of genes controlling a trait increases, progressively more progeny are needed per backcross family to retain enough of the resistance alleles through successive generations while decoupling them from alleles that otherwise define Chinese chestnut characteristics. Despite the potential for refining estimates of the needed number of progeny as backcrossing proceeds (Burnham et al., 1986), complications due to inter-species chromosomal incompatibilities, such as translocations, inversions and reduced recombination, can result in the need for even larger progeny sizes, further emphasizing the need to understand the genetic architecture of the trait.

Quantitative Trait Locus (QTL) mapping studies were initiated to test the hypothesis of the two-gene model for blight resistance. The initial studies focusing on one inter-species F2 hybrid family suggested that possibly as few as three QTLs were involved (Kubisiak et al., 1997, 2013). However, recent analyses of backcross progenies suggests that although later generation trees often present classic American chestnut phenotypes, the level of blight resistance is not as high as that of Chinese chestnut (Cipollini et al., 2017; Steiner et al., 2017; Clark et al., 2019). These results challenge the wisdom of backcross breeding for blight resistance by implying that the genetic models of a limited number of major loci may be oversimplified.

Results from a recent study evaluating the potential for genomic selection in the TACF breeding program (Westbrook et al., 2020) suggested that blight resistance was polygenically inherited (i.e., a quantitative trait), thus complicating the decoupling of resistance alleles from alleles of other traits and the recovery of resistant trees with otherwise American chestnut characteristics. Additionally, it is entirely possible that the genes conferring blight resistance in Chinese chestnut are complicit in the biology of traits (morphological, phenological, eco-physiological) that differentiate the two species. Therefore, to predict and track the success of the American chestnut restoration breeding strategy, it will be helpful to not only resolve the genetic architecture of the resistance trait but also to determine whether QTLs for resistance are the same as or overlap with those impacting species defining traits. In addition, it is important to know the genomic locations of the QTLs for blight resistance relative to those for *Phytophthora* root rot (PRR) resistance (Santos et al., 2017; Zhebentyayeva et al., 2019), as resistance to both diseases will be necessary for successful restoration over the species range (Westbrook et al., 2019).

Although single-family QTL mapping studies have significant power to detect marker associations with traits, they can only detect loci that are segregating in the two parents. Most likely, families having different parental/ancestral sources of resistance genes will give different results. Therefore, to draw a more complete picture of the genetic architecture of the resistance trait at the species level, it is important to test different sources of resistance for shared QTLs as well as loci segregating in one cross and not another. In contrast to single-family QTL mapping, genome-wide association studies (GWAS) provide an approach that does not require associations to be tracked within families, but instead takes advantage of linkage disequilibrium between markers and traits in populations to identify QTLs.

In combination, GWAS and single-family-QTL mapping approaches can potentially provide a robust evaluation of the genetic architecture of important traits. For this reason, we performed a series of GWAS studies for blight resistance using multiple backcross families where in each family the original resistance donor was one of three unrelated Chinese chestnut trees (Mahogany, Nanking or M16) and a QTL study on a Mahogany-derived F2 family. We evaluated trees for resistance to blight and nine morpho-phenological (M/P) traits that are the hallmarks of species differentiation between American and Chinese chestnuts (Hebard, 1994, 1995). The genomic locations of the blight resistance and the M/P trait QTLs were compared as were the locations of PRR resistance QTLs identified previously (Santos et al., 2017; Zhebentyayeva et al., 2019). In addition, we attempted to identify candidate genes (CGs) controlling blight resistance and M/P traits by two approaches: a) utilizing previously published transcriptomic data of canker and healthy stem tissues (Barakat et al., 2009, 2012) to identify the differentially expressed genes that are located within the blight resistance QTLs, and b) BLASTing the sequences of the most significant markers in the M/P QTLs against *C. dentata* genome v1.1 (Westbrook and Schmutz, 2020) to delimit genomic regions underlying the M/P traits and identifying genes in these regions having *Arabidopsis* homologs with relevant putative functions using the highly-curated *Arabidopsis* protein sequence database (https://bar.utoronto.ca/thalemine).

## Materials and methods

### Mapping families

**Figure 1:**
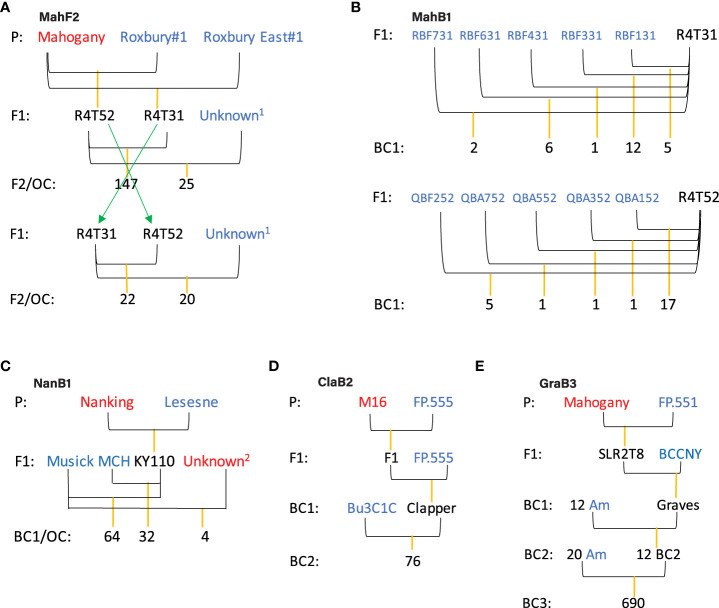
Pedigree of families used for trait mapping. For each cross, the female parent is drawn on the left, the male parent on the right. The red colored varieties are Chinese chestnuts (Mahogany, Nanking, and M16) serving as blight resistance donors. The blue colored parents are American chestnut trees. Each F1 hybrid of Chinese chestnut and American chestnut either was crossed with another F1 sibling to produce a F2 family **(A)**, or “backcrossed” with one or multiple American chestnut trees to produce backcross families **(B–E)**. The term “backcross” is not used in literal sense since different American chestnut individuals are typically used in each generation to conserve genetic diversity in progeny. OC, outcross siblings as maternal siblings with unknown pollen source. **(A)** MahF2, F2 family composed of 214 F2 or outcross siblings with the blight resistance source Mahogany. The pedigree is the same as used by Kubisiak et al. (1997). Unknown^1^ is an unknown American chestnut pollen source. **(B)** MahB1, backcross 1 family composed of 51 siblings with the resistance source Mahogany. **(C)** NanB1, backcross 1 family composed of 96 siblings with the resistance source Nanking and 4 outcross siblings with unknown Chinese chestnut resistance source. Unknown^2^ is an unknown Chinese chestnut pollen source. **(D)** ClaB2, backcross 2 family composed of 76 siblings with the same resistance source M16. **(E)** GraB3, backcross 3 family composed of 690 siblings with the same resistance source Mahogany. For simplicity, “Am” in BC3 family pedigree was used to denote American chestnut trees used as female parents. Note, currently there is uncertainty about the ancestry of tree “Graves” and we adopt the original pedigree and family name in this study.

The pedigrees of the five families we studied are depicted in **Figure 1**. Three unrelated Chinese chestnut genotypes (names in red) were used as blight resistance donors, whereas 52 American chestnut genotypes (names in blue) were used as recipients (recurrent parents) in different generations to reduce inbreeding and increase the number of progenies (family sizes). Note that all parents (Chinese and American chestnuts and their hybrids) are highly heterozygous and mostly unrelated, so these are not traditional F2 and backcross (BC) crosses. “Mahogany” is the resistance donor for Mahogany F2 (MahF2, 214 trees including reciprocal cross and outcross siblings), Mahogany BC1 (MahB1, 51 trees), and Graves BC3 (GraB3, 690 trees) families; “Nanking” is the resistance donor for Nanking BC1 (NanB1, 96 trees) family; and “M16” is the resistance donor for Clapper BC2 (ClaB2, 76 trees) family. The NanB1 family contains four outcross seedlings derived from one American chestnut genotype “Musick” open pollinated by one or more Chinese chestnut genotypes “opMusick” (name in red).

The GraB3 family is the result of 24 crosses involving 20 American chestnut trees as the seed parents and 12 BC2 trees as the pollen parents. Each cross resulted a variable number of seedlings ranging from 5 to 80. Note, there is uncertainty about the grandparent tree “Graves” being BC1 (**Figure 1E**) or F1 (**Supplementary Figure 1**). Here, we adopt the original pedigree and family name, with the recognition that these may be changed later with more evidence. However, the pedigree issue has, if any, a negligible effect on our mapping study.

In total, 1131 trees (seedlings grown from seed) from the five families were phenotyped and/or genotyped. Based on the genetic complexity and availability of phenotypic data for the individual families, different subsets of these trees were used in the following studies:

a) 147 full-sibs in MahF2 family, derived from the cross in the same direction (**Figure 1A**), were used for genetic map construction and multiple QTL mapping (MQM) of blight resistance. These trees include 83 siblings from a cross made in 1991 that were used in previous mapping studies (Kubisiak et al., 1997, 2013), plus 64 from an identical cross made in 2009 to expand the population for genetic mapping.
b) The pool of four backcross families, as well as each family separately, was used in GWAS for blight resistance. In total, 1094 trees were used for these studies.
c) Various numbers (185 to 965, **Supplementary Table 1**) of trees were used in GWAS for nine M/P traits.

### Blight resistance assessment

Sibling trees used in this study were generated by breeding efforts conducted over nearly 20 years at TACF’s Meadowview Research Farms in cooperation with the Connecticut Agricultural Experiment Station and Pennsylvania State University. All seeds were generated through controlled pollination in one year and planted in the following year. Seeds for the MahB1, NanB1, and GraB3 families were each planted in one year at one location, while seeds for the MahF2 and ClaB2 families were planted in two different years, respectively, but at the same location (**Supplementary Table 2**). Seeds from the same families planted at the same time at the same location are referred to as cohorts. In all, seven cohorts for the five families were planted in completely randomized designs and evaluated for blight resistance.

Two to four-year-old seedlings were artificially inoculated in June of the respective years with two blight fungal strains, SG2-3 and Ep155, using the cork borer-agar disk method described by Griffin et al. (1983). Inoculum of the more virulent strain, Ep155, was applied to the lower, larger portion of the main stem to prolong tree life during the first year of canker expansion, whereas SG2-3 inoculum was applied higher up on the stem. Inoculations were made on the same side of a tree, which was either the north side in east-west-running rows, or the east side in north-south-running rows. The locations of the inoculations were kept consistent to minimize environmental variation. In general, one inoculation per tree was made with each strain. One exception was that most four-year-old trees (**Supplementary Table 2**) were inoculated twice with each strain. Another exception was that the second cohort of 64 MahF2 trees were inoculated three times with each strain; with the successive inoculations being placed with one-third turn of a spiral around the stem; also, in this case, the positions of the two strains were randomized.

The length and/or width of cankers caused by each strain were measured in the same summer and/or fall/winter of the inoculations (**Supplementary Table 2**). For each tree, the canker lengths and widths were recorded separately by strain (SG2-3 or Ep155) and time of measurement (“early or “late” season). Here, “early” refers to the time of evaluation when cankers are still actively growing (earlier in the season), while “late” refers to the time when canker expansion slows down or stops (later in the season) due to either colder autumn temperatures and/or shorter days (in the cases of B1 and B2 families) or encircling of the stems by cankers (in the cases of MahF2 and GraB3). In the GraB3 family, the canker size was evaluated relatively early in the season (August) when the largest cankers had already encircled the stems and could no longer expand in width. For this reason, canker size of the GraB3 family was only evaluated once and treated as the “late” measurement.

The canker size of B1 and B2 trees was computed as (length + width)/2. But in GraB3 family, only the width measurement was used, and in MahF2 family, only the length measurement was used, due to the availability of only width or length data in the respective cohorts. In cases of two or three inoculations of one strain, the average of canker sizes over inoculations of the strain was used.

Prior to data integration across cohorts, the canker size data of each cohort were normalized by the z-score method using the formula: $z=(x-\bar{x}$ )/s, where *z* is the normalized canker size, *x* is the raw canker size, $\bar{x}\;$ and *s* are the mean and standard deviation of canker size in the cohort indexed by strain and time. The inclusion of two factors each with two categories results in four dimensions of canker size data (SG_Early, SG_Late, Ep155_Early, Ep155_Late, where SG refers to SG2-3).

### Evaluation of morpho-phenological traits

In addition to blight resistance, nine M/P traits were evaluated. Among these, tree height and stem diameter were measured on a continuous scale, while the remaining seven traits were scored on categorical scales even though some of them (e.g., leaf emergence) are in fact continuously distributed. In general, the trees with no leaf hair, slight amount of vein hair, late leaf emergence time, male fertility, cylindrical bud shape, erect tree form, and strong central leader were considered as American chestnut type; the trees with leaf hair, high amount of vein hair, early leaf emergence time, male sterility, round bud shape, spreading tree form, and weak central leader were considered as Chinese chestnut type. For leaf emergence and vein hair, we also scored an intermediate type.

Height data were collected in all backcross trees and some of the F2 trees. In the B1, B2 and F2 families, height was measured for two- or three-year-old trees; while in the B3 family, it was measured for six-year-old trees. Diameter data were measured for some trees in the B2, B3 and F2 families. In B2 and F2 families, diameter was measured for two- or three-year-old trees; while in B3 family, it was measured for eight-year-old trees (**Supplementary Table 2**). Height and diameter data of each cohort were individually normalized using the z-score method and then pooled together.

Leaf hair, vein hair, and bud shape data were collected for all B1 and B2 trees and some of the F2 trees; male sterility, tree form, and central leader data were collected for all trees in only the B1 and B2 families. Male sterility was scored as anthers exserted (fertile) or not (sterile). Sterile trees were rerated later in the season to make sure they had not just exserted late. For each of these traits, if a tree resembled American chestnut, it was assigned a value “0”. Otherwise, it was assigned a value “1” indicating a stronger resemblance to Chinese chestnut. For vein hair, an additional value of “0.5” was assigned to a tree if it had an intermediate amount of vein hairs.

Leaf emergence was evaluated for all trees in the backcross families and some of the trees in the F2 family. The trees were scored as “emerged” or “not emerged” on a cutoff date (in either late April or early May) for each cohort. A tree was rated “emerged” if the internodes were visible or buds had been killed by a spring frost. The score was then converted to “0” (not emerged) or “1” (emerged). The B1, B2 and F2 trees were scored only once, while the B3 trees were scored twice on May 1^st^ and 7^th^ in the same year. The score of a B3 tree is the mean of two evaluations.

For the detailed description of the M/P traits in American or Chinese chestnuts and phenotyping time, see **Supplementary Tables 1** and **2**.

### Correlation analysis

Pairwise correlations were analyzed between the canker size in four dimensions (SG_Early, SG_Late, Ep155_Early, and Ep155_Late) and the nine evaluated M/P traits (**Supplementary Data 1**) using the “rcorr” function of R package “Hmisc”. A matrix of Pearson’s correlation coefficients (r) for all possible pairs of traits was computed, as well as a matrix of significance (p) for each r value. R package “corrplot” was used to draw the correlation matrix plot (correlogram).

### SNP marker development and genotyping of mapping families

Single nucleotide polymorphisms (SNP)s were identified from Unigene assemblies *C. dentata* AC454_v3 and *C. mollissima* Call_v2 (Barakat et al., 2012), using the method described by Kubisiak et al. (2013), except that multiple SNPs were allowed from the same EST contig to increase the chance of getting high quality markers. SNPs identified from AC454_v3 assembly were named as “AC454v3c”, followed by the number of contig it is from, “_”, and its base pair position in the contig. SNPs from CCall_v2 assembly were named in the same style with the beginning of “CCallv2c”.

The AC454v3c and CCallv2c SNP markers were interrogated on an Illumina Infinium BeadArray platform (Illumina) for 1131 sibling trees in all five families. The genotyping data were analyzed using the genotyping module of GenomeStudio® software v2011.1 (Illumina Inc., San Diego, CA, USA). Genotypes with a GeneCall score < 0.15 were set as missing values.

In addition to the sibling trees, three Chinese chestnut genotypes (Mahogany, Nanking, Vanuxem) and 13 American chestnut parents for GraB3 siblings were genotyped and analyzed using the two sets of SNP markers described above.

### Hapmap file preparation

In total, 4451 AC454v3c or CCallv2c SNPs were genotyped in 1131 trees. We discarded the monomorphic markers and formatted the genotypes of the remaining 3920 SNPs as a Hapmap file (**Supplementary Data 2**). The position of each SNP in *C. mollissima* (Vanuxem) reference genome assembly v4.3 (Carlson and Staton, 2014; Staton et al., 2020) was used as its position (“chr” and “pos”) in the Hapmap file.

To find the genomic position of SNPs, EST sequences harboring these SNPs were BLASTed against the genome assembly with NCBI BLAST software (downloadable at ftp://ftp.ncbi.nlm.nih.gov/blast/executables/blast+/LATEST). From the BLAST result for each SNP, we determined its genomic location in scaffold/chromosome, and position in cM on the reference genetic map used for pseudochromosome assembly (Staton et al., 2020). The order of chromosomes from 1 to 12 in this study corresponds to linkage groups (LG) A to L reported in previous publications (Kubisiak et al., 1997, 2013). The SNPs without a BLAST hit were assigned to group “0”.

For simplification, we used the reference genetic map position (cM) for each SNP marker. The cM positions provide sufficient mapping resolution for our GWAS study. Readers interested in base pair positions for any subset of markers may use the provided EST sequences (**Supplementary Data 3**) that harbor those markers to BLAST against any available chestnut genome (e.g. using the Phytozome portal at https://phytozome-next.jgi.doe.gov/).

### Population structure and GWAS

Principal component analysis (PCA) for the 1131 trees was performed with TASSEL 5.2 software (Bradbury et al., 2007) to reveal possible stratification of the population used for GWAS. For continuously and categorically scored traits, we performed GWAS with TASSEL 5.2 and GAPIT 3 software (Tang et al., 2016), respectively. In both cases, the “PCA + Kinship” method was used with the mixed linear model (MLM) function. The first five principal components (PC) were included in the MLM model as fixed effects to account for population structure. The kinship matrix was used to account for genetic relatedness between individual trees. For GWAS of blight resistance using combined canker size data, two factors (time of evaluation and strain of fungus) were treated as additional fixed effects in the MLM model. For GWAS of blight resistance using only SG2-3 or Ep155-induced canker size data, only time of evaluation was treated as additional fixed effect.

For blight resistance, we performed GWAS with the pool of four backcross families, as well as with each backcross and F2 family separately. For the nine M/P traits, the trait data were collected in different subsets of trees (**Supplementary Tables 1, 2**), so different numbers of trees were used depending on the trait being analyzed.

Prior to PCA with all trees or each GWAS, the SNP data were filtered using TASSEL. Firstly, a subset SNP dataset of the trees to be used was made from the original Hapmap file. Secondly, the subset dataset was filtered to further discard those SNPs missing genotypes in > 20% of the trees or having a minor allele frequency < 1%. Finally, the filtered dataset was used to generate the PCs and kinship for the subsequent population structure or MLM analysis.

Considering the potentially large linkage disequilibrium blocks due to relatedness of sibling trees in this study, the QTL intervals were reported in addition to the most significant SNPs. To reduce the chance of missing meaningful SNPs, we adopted a relatively relaxed 100 × Bonferroni corrected p-value threshold. Since the number of SNPs used in each GWAS varied over a large range after filtering (1060 to 2229, **Supplementary Table 1**), for simplicity, we calculated a cutoff p-value 0.0022 based on the largest SNP number (2229). Any SNP with a p < 0.0022 was considered significant, while SNPs with a p < 0.005 in the vicinity of a significant SNP were used to delineate the QTL boundaries. The GWAS plots were drawn with R package “qqman”.

Where two or more GWAS analyses detected QTLs in overlapping intervals, we declared the region spanning all the overlapping intervals a composite QTL interval.

### QTL mapping in MahF2 family

A genetic map was constructed with the SNP markers described above plus a panel of simple sequence repeat (SSR) markers derived from EST contigs of Chinese and American chestnuts (Kubisiak et al., 2013). The SSRs were assayed for these trees as described in Kubisiak et al. (2013). For genetic map construction and QTL mapping only the core set of MahF2 progeny (147 trees over the two cohorts) were used, those being derived from the R4T52 x R4T31 cross (**Figure 1A**). Genetic maps were constructed using JoinMap 4.1, default parameter settings, and the regression algorithm (Van Ooijen, 2006). Parental genotypic data for 18 SNPs were insufficient to determine segregation type. Each of the 18 SNPs was coded as two possible segregation types and treated as unrelated markers with the expectation that only the correctly coded one would be mapped (and the other excluded); when both versions mapped, both were excluded.

Trees or markers with > 40% missing data were excluded, and additional markers were excluded if they had both distorted segregation ratios (p < 0.0001) and > 20% missing data. In addition, redundant markers (having nearly “identical” genotypic data with other markers) were excluded during map construction but were assigned to appropriate map locations when possible. Other distorted markers were initially included in map construction, but isolated, distorted markers on the map were removed from the genotypic data, and the maps recalculated; this sometimes necessitated several rounds of recalculation to remove additional isolated, distorted markers unmapped in earlier rounds. The resultant map was compared to an updated Chinese chestnut reference genetic map (Staton et al., 2020, **Supplementary Data 4**). If a marker was mapped to a substantially different position, a lower-case letter is added to the end of the marker name in the F2 map.

QTL mapping was performed using MapQTL 6 (Van Ooijen, 2009). Markers with the same heterozygous genotype for both parents (segregation type <hkxhk>) were excluded from the analysis to facilitate calculation. Permutation tests (1000 iterations) were used to determine the critical (p = 0.05) genome-wide logarithm of the odds (LOD) value for interval mapping and MQM. Cofactors for MQM were chosen based on the results of interval mapping and MQM runs, plus automatic cofactor selection. Automatic cofactor selection derived cofactors were dropped from MQM models if they were not associated with significant LOD peaks. MQM analyses were limited to those dimensions of canker size data for which significant LOD peaks were detected by interval mapping. The LOD profile was separately computed for 1991, 2009, and combined cohorts. The percentage of phenotypic variance explained by QTLs in 1991 and 2009 cohorts were also separately computed.

### Comparing QTL results for blight resistance and *Phytophthora* root rot resistance

The blight resistance QTLs detected by GWAS were mapped to the *C. mollissima* reference genome v4.3 through the reference genetic map embedded in the genome assembly (Carlson and Staton, 2014; Staton et al., 2020), while the QTLs detected by each MQM were placed on a genetic map constructed with the specific mapping population. To facilitate the comparison of the QTL intervals detected by different methods and resistance traits, the SSRs and SNPs in MQM detected intervals for blight resistance and for PRR resistance in two previous studies (Santos et al., 2017; Zhebentyayeva et al., 2019) were mapped to the *C. mollissima* reference genome using the same method described in Hapmap file preparation section above.

### Transcriptome analysis for mining blight resistance candidate genes

The RNAseq data of healthy and cankered stem tissues of American and Chinese chestnut trees used in previous transcriptome studies (Carlson, 2008; Barakat et al., 2009, 2012) were reanalyzed to evaluate the potential roles of the QTL interval genes on blight resistance. The sequencing reads were aligned to the *C. mollissima* reference genome assembly v4.3 with the STARlong function of STAR aligner software (Dobin et al., 2013). Uniquely mapped reads at the gene level were quantified with HTSeq software (Anders et al., 2015). Raw counts were subjected to differential expression analysis by the R package ‘edgeR’ (Robinson et al., 2009). A likelihood ratio test (LRT) among two canker samples and eight healthy samples was performed to identify differentially expressed genes (DEG). Genes with a fold change > 2 and an adjusted p < 0.05 were considered as significant DEGs. The list of DEGs was sorted by their positions in the *C. mollissima* reference genome. Only those DEGs residing in blight resistance QTL intervals (detected by GWAS or MQM) were considered CGs. The putative functions of the CGs were explored by BLASTing their protein sequences against *Arabidopsis* protein sequences from the TAIR10 genome annotation release (Berardini et al., 2015) with an e-value cutoff of 1e^-5^ and only the best hit being retained.

Following this, we selected a high-priority list of CGs based on their close linkage to the most significant markers (SNP or SSR with the lowest p value in GWAS or highest LOD in MQM) in their respective QTLs. Those CGs within 0.5 cM (1 cM window) of the QTL’s most significant markers were regarded as the high-priority CGs and subject to literature search to explore the implications for disease resistance of their homologs in other plant species. Also, to provide markers for potential use in marker assisted selection (MAS), we made a list of the significant markers either with p < 0.005 in GWAS-detected QTLs or within 1 LOD interval of the MQM-detected QTLs.

### Candidate gene mining for morpho-phenological traits

We used the Phytozome plant genomics portal (Goodstein et al., 2012) and recently released *C. dentata* genome v1.1 (Westbrook and Schmutz, 2020) to characterize the genomic regions associated with the mapped M/P traits. The EST sequences encompassing the SNPs in the QTLs were BLASTed against the *C. dentata* genome. Two or more BLAST hits on the same gene were only counted as one non-redundant hit, and genomic regions with ≥ 3 non-redundant hits were further explored. For these regions, the gene content was extracted from the *C. dentata* genome annotation file Cdentata_673_v1.1.defline.txt (Westbrook and Schmutz, 2020) and multiple protein sequence alignments were conducted using EMBL-EBI sequence analysis tools (McWilliam et al., 2013) and visualized with Jalview 2.11.2.6 (Waterhouse et al., 2009).

## Results

### Population structure

**Figure 2:**
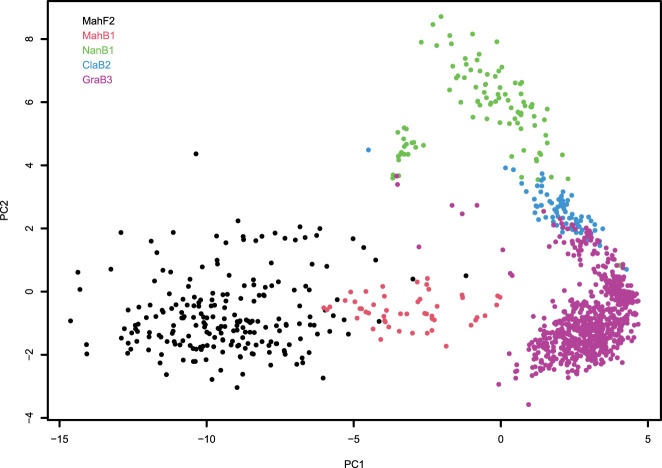
Clustering of chestnut trees used in trait mapping studies as illustrated by the plot of first and second principal components (PC1 and PC2) computed with genotypic data of 2244 SNPs. Each circle on the plot represents a hybrid-origin chestnut tree. Trees from different families are differentiated by different colors.

The PCA with 2244 SNP markers after filtering and all 1131 trees revealed the existence of population strata and in general, confirmed family identities at the genetic level. The plot of the first and second PC revealed that trees in each family tend to aggregate together, although among families, there are no clear boundaries (**Figure 2**). The first PC (PC1) accounts for 20.7% of the genotypic variance and reflects the variation of the genomic content of American chestnut in the trees; with increasing PC1 values, the theoretical percentage of American chestnut genome increases from ~50% in the F2 family to ~93.75% in the B3 family. The second PC (PC2) accounts for 4.3% of the genotypic variance and reflects the different Chinese chestnut genome donors. The descendants of “Nanking” (NanB1 family) have the highest PC2 values, those of “Mahogany” (MahF2, MahB1, GraB3 families) have lowest PC2 values, and those of “M16” (ClaB2 family) have PC2 values in between. In addition to the clustering of families, the spatial distribution of each family appears different, specifically showing higher diversity in the F2 family than in the B3 family (**Figure 2**) as would be expected given the additional backcrossing to American chestnut in the B3 family.

### Phenotypic variability and correlation analysis

**Figure 3:**
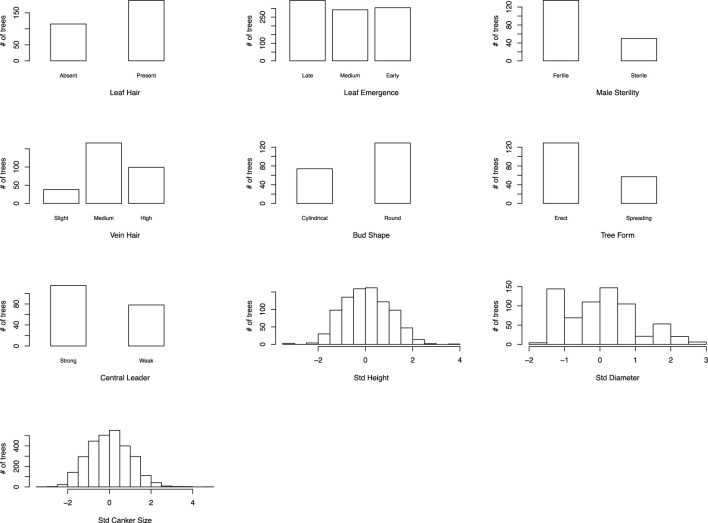
Distribution of integrated canker size (inverse representation of blight resistance) data and nine morpho-phenological traits. For seven morpho-phenological traits, trees resembling American chestnut were grouped on the left and trees resembling Chinese chestnut on the right. For leaf emergence and vein hair, an additional “medium” group was made to reflect that some trees had intermediate phenotypes. Std, standardized.

For this study, crosses were made, and progeny trees inoculated and evaluated over a timespan of two decades in two locations (**Supplementary Table 2**). We evaluated blight resistance (i.e., measured canker size after artificial inoculation) for each tree in each cohort during the first few months of the growing season, since experience has shown this to be a highly correlative measure of resistance over the longer term (FV Hebard, unpublished). After data normalization and integration across cohorts, the canker size data displayed a normal distribution across the 1094 trees (**Figure 3**).

In addition to canker size, nine M/P traits with distinct characteristics in American and Chinese chestnuts were evaluated. After data normalization and integration, both tree height and stem diameter showed an approximate normal distribution as expected for quantitative traits. For seven categorically scored traits, the ratio of American chestnut vs. Chinese chestnut-like trees varied drastically and no obvious or consistent distribution pattern was identified (**Figure 3**).

**Figure 4:**
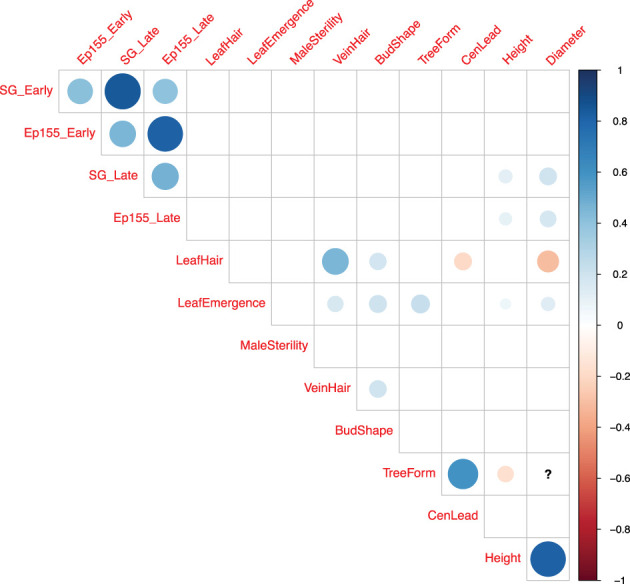
Correlogram (correlation matrix plot) of four canker size measurements and nine morpho-phenological traits. Positive correlations are displayed with blue dots, while negative correlations are displayed in red. The strength of correlation is illustrated both in intensity of color and size of dots. The correlation coefficients having p-values larger than 0.05 are considered as not significant and not shown. The question mark in the figure indicates that there are not enough data to compute that correlation coefficient.

Pairwise correlation analyses were performed in four different dimensions (SG_Early, SG_Late, EP155_Early, EP155_Late) of canker sizes plus nine M/P traits. The magnitude and direction of correlations between traits are shown in **Figure 4** and listed in **Supplementary Table 3**. Among the four dimensions, strong positive correlation exists between early- and late-assessed SG2-3-induced canker sizes (r = 0.85), as well as between early- and late-assessed Ep155-induced canker sizes (r = 0.82), but not in the other pairs of canker size data (r = 0.40 - 0.47). The lack of strong correlations between SG2-3 and Ep155-induced canker sizes indicates the relative independence of canker growth caused by the two strains even though they were inoculated at the same time on the same trees. Prior to normalization, we found that Ep155 induced larger cankers across all cohorts as expected.

No strong correlations were found between canker size and individual M/P traits. A few significant Pearson coefficients (r) are illustrated in **Figure 4** for the correlations between canker size and tree height or stem diameter. However, the absolute r values fall below 0.3 suggesting that the correlations are weak (**Supplementary Table 3**). Among the other traits, we found a strong, positive correlation (r = 0.82) between tree height and stem diameter; and a moderate, positive correlation (r = 0.6) between tree form and central leader. We also observed a moderate, positive correlation (r = 0.46) between leaf hair and vein hair; and a weak, negative correlation between leaf hair and diameter (r = -0.3) (**Figure 4**, **Supplementary Table 3**).

### GWAS for blight resistance

GWAS for blight resistance was performed with the pool of all four backcross families, and with each backcross family and the F2 family separately.

**Figure 5:**
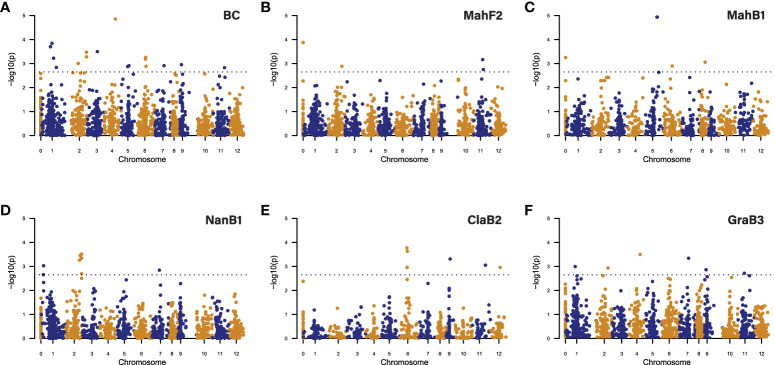
Manhattan plots showing significant SNP/QTLs detected by GWAS analysis of blight resistance in chestnut using **(A)** BC as all backcross families, **(B)** MahF2 family, **(C)** MahB1 family, **(D)** NanB1 family, **(E)** ClaB2 family, **(F)** GraB3 family. In each plot, each dot represents a SNP. The X-axis shows the positions of SNPs in the Chinese chestnut reference genome v4.3. Numbers “1” to “12” on the X-axis represent 12 chromosomes in the chestnut genome. Number “0” represents the unmapped SNPs. The Y-axis shows the significance level (negative base 10 logarithm of p value) for each SNP tested. The dashed horizontal line represents the genome-wide significance threshold adopted in this study. SNPs above the threshold line are significant and considered for their potential in delineating QTLs.

Treating the strain of fungus as an environmental factor (SG2-3 vs. Ep155), we first performed GWAS using canker size data induced by both strains (combined strain analysis), as this would likely detect QTLs associated with general (non-strain specific) blight resistance. The multi-family GWAS detected 10 significant SNPs (i.e., peak SNPs for 10 QTLs) on chromosomes 1, 2, 3, 4, 5, 6, 7, 9, and 11 (**Figure 5A**). Seven of these SNPs were also detected in one or two single-family GWAS; the other three were missed in all single-family GWAS. However, when the significance threshold was relaxed to p = 0.05, two additional QTLs were detected in single-family GWAS (**Figure 5**, **Supplementary Table 4**).

GWAS in the GraB3 family, with a more complex genetic background compared to the other families, detected six QTLs, while the other single-family GWAS only detected two to four QTLs per family. Collectively, the five single-family GWAS detected 15 QTLs, including eight that multi-family GWAS failed to detect. Altogether (multi- and single-family analyses), GWAS detected 18 peak SNP/QTLs distributed on 11 of the 12 chestnut chromosomes, while no QTLs were detected on chromosome 10 (**Figure 5**, **Supplementary Table 4**).

Each significant GWAS-detected SNP has a relatively small R^2^ value, indicating their small effect on canker size. In the multi-family GWAS QTLs, the most significant SNPs (peak SNPs) explain only 0.57 - 1.2% of the total phenotypic variance. In the single-family GWAS QTLs, the peak SNPs explained 0.73 - 6.81% of the total phenotypic variance in the respective families (**Supplementary Table 4**).

We then performed GWAS using SG2-3 or Ep155 induced canker size data (separate strain analysis), as this could potentially detect additional QTLs associated with resistance to specific fungal strain(s).

GWAS with strain specific canker data detected 26 QTLs, 10 of them were detected with both strain specific datasets, three detected with only the SG2-3 dataset, and 13 detected with only the Ep155 dataset. Unlike for the combined strain analysis, single-family GWAS with the NanB1 family detected the highest number (18) of QTLs among all strain specific analyses, while multi-family analysis detected the second highest number (12) of QTLs (**Supplementary Table 5**).

Similar to the combined strain analysis, multi-family GWAS with the strain specific datasets detected QTLs with small marker R^2^, explaining only 1.08 - 1.85% of the phenotypic variance. However, single-family GWAS detected QTLs with more variable marker R^2^, explaining 1.65 - 19.81% phenotypic variance (**Supplementary Table 5**).

Among 18 QTLs detected by GWAS using combined data, 15 have overlapping intervals with the QTLs detected by GWAS using strain specific data. In total, GWAS detected 29 QTLs for blight resistance across 12 chromosomes (**Supplementary Figure 2**, **Supplementary Tables 4, 5**).

### MQM for blight resistance

The core subset of the MahF2 family, composed of 147 trees in two cohorts (83 and 64 trees, respectively), was used for construction of a F2 linkage map and subsequent MQM mapping. The genotypic data of this subset comprised 111 SSRs and 1113 SNPs after filtering.

Ten LGs were defined at LOD 15, the other two (corresponding to chromosomes 4 and 6) at LOD 25, resulting in a 706-marker map (74 SSRs, 632 SNPs) with a total map length of 830.1 cM in 12 LGs. Additionally, 61 redundant markers were assigned to map positions, raising the total number of mapped markers to 767 (**Supplementary Table 6**). Seven of the 12 LGs displayed moderate (chromosomes 1, 7, 8, and 10), localized (chromosome 9), or very little segregation distortion (chromosomes 3 and 4). The remaining LGs showed more extensive segregation distortion. More than 50% of the non-redundant markers (422) were of segregation type <hkxhk>; less than 5% (33) of fully informative types (<abxcd> or <efxeg>); while the others segregated with two alleles in one (146) or another (105) F1 parent (<lmxll> or <nnxnp> type).

Interval mapping only detected one significant QTL on chromosome 2 for SG_Early and Ep155_Late canker sizes. MQM found no additional QTL for the SG_Early data; however, for the Ep155_Late data, QTLs were identified on five additional chromosomes: 5, 7, 8, 11, and 12. All six QTLs were significant in the 2009 and combined cohorts; QTLs on chromosomes 2, 7, and 8 were also significant in the 1991 cohort, although the peak position of the QTL on chromosome 8 was shifted. QTLs on chromosome 2, 5 (in 2009 cohort only), 7, and 8 accounted for large percentages of the phenotypic variances in a six-cofactor QTL model (**Table 1**).

**Table 1:** Multiple QTL Mapping results for blight resistance with the expanded F2 interspecific hybrid population (resistance source *C. mollissima* ‘Mahogany”).

Canker Size	Chr	Cofactor	Pos	Interval	Var%		LOD		
					1991	2009	1991	2009	Combined
SG_Early	2	AC454v3c8420_390	29.894	29.887 - 45.916	10.9	28.0	ns	4.21	6.16
Ep155_Late	2	AC454v3c8420_390	29.894	29.887 - 45.916	10.4	9.6	4.15	5.19	9.34
	5	AC454v3c8144_537	NA	21.391 - 22.159	3.7	15.4	^1^ns	7.5	9.08
	7	AC454v3c7956_697	34.355	34.355 - 47.929	14.6	13.1	5.58	6.64	12.22
	8	^3^AC454v3c16666_1001	6.881	6.881 - 23.188	10.8	^2^na	4.64	ns	7.38
	8	CmSI0710	16.423	10.067 - 23.188	na	26.9	ns	11.12	14.18
	11	CCallv2c25306_1210	27.996	26.454 - 31.019	5.5	8.1	ns	4.53	6.85
	12	AC454v3c16632_906	27.978	27.978 - 30.71	8.5	8.3	ns	4.59	8.06

Canker Size, canker size data with the names reflecting the fungal isolate (SG2-3 and Ep155) and evaluation time (early and late); Chr, chromosome; Cofactor, SNP or SSR marker that best represents a QTL; Pos, the position (cM) on the reference genetic map embedded in C. mollissima (Vanuxem) reference genome assembly v4.3; Interval, 2-LOD support interval of QTL in cM; Var%, the percentage of phenotypic variance explained by a QTL; LOD, logarithm of odds; ^1^ns, not significant; ^2^na, not available. ^3^AC454v3c16666_1001 is not a cofactor, but included here to capture the significant QTL on chromosome 8 for the 1991 cohort.

### GWAS for morpho-phenological traits

Most of the categorically scored M/P traits did not show a 3:1 phenotypic ratio in the F2 or 1:1 in the backcrosses as is typical of a monogenic, dominant trait. For this reason, we refer to the GWAS-detected loci as QTLs.

**Figure 6:**
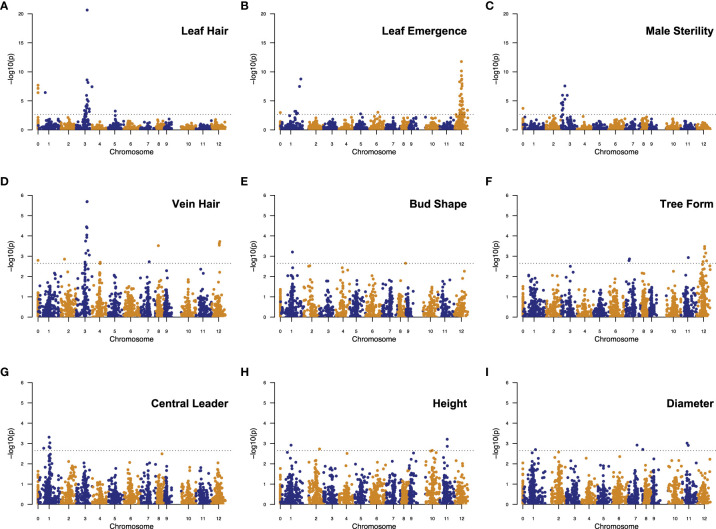
Manhattan plots showing significant SNPs/QTLs detected by GWAS analysis of nine morpho-phenological traits in chestnut: **(A)** Leaf Hair, **(B)** Leaf Emergence, **(C)** Male Sterility, **(D)** Vein Hair, **(E)** Bud Shape, **(F)** Tree Form, **(G)** Central Leader, **(H)** Height, **(I)** Diameter. In each plot, each dot represents a SNP. The X-axis shows the positions of SNPs in the Chinese chestnut reference genome v4.3. Numbers “1” to “12” on the X-axis represent 12 chromosomes in the chestnut genome. Number “0” represents the unmapped SNPs. The Y-axis shows the significance level (negative base 10 logarithm of p value) for each SNP tested. The dashed horizontal line represents the genome-wide significance threshold adopted in this study. SNPs above the threshold line are significant and considered for their potential in delineating QTLs.

GWAS on each categorical trait, except for bud shape, produced one highly significant QTL peak and some other significant SNPs with the p-values over a large range (**Figure 6**). For these traits, we ignored those marginally significant SNPs and focused on the most significant QTLs with clustered (genetically linked) significant SNPs. For leaf hair and vein hair, we also considered a few highly significant single SNPs. Given this, we found 19 SNP/QTLs associated with nine M/P traits, located on chromosomes 1, 2, 3, 7, 10, 11, and 12 (**Supplementary Table 7**).

Bud shape, central leader, tree height, and stem diameter have overlapping or adjacent QTL intervals in a narrow region, 40.12 - 48.56 cM, on chromosome 1. Leaf hair and vein hair share a QTL peak on chromosome 3. Leaf emergence, vein hair, and tree form have overlapping QTL intervals on chromosome 12 (**Figure 6**, **Supplementary Table 7**).

The categorical trait QTLs determined by GWAS generally showed larger effects than those for the continuously distributed traits such as tree height and stem diameter. The peak SNPs for leaf hair and male sterility QTLs, both on chromosome 3, explained 18.65% and 13.71% of the phenotypic variance for each trait, respectively. The peak SNPs of the other categorical trait QTLs explained from 2.95 - 5.93% of the phenotypic variance. Comparatively, the peak SNPs of the height and diameter QTLs explained only 1.20 - 2.04% of the phenotypic variance (**Supplementary Table 7**).

### Comparison of blight resistance mapping by MQM and GWAS

**Figure 7:**
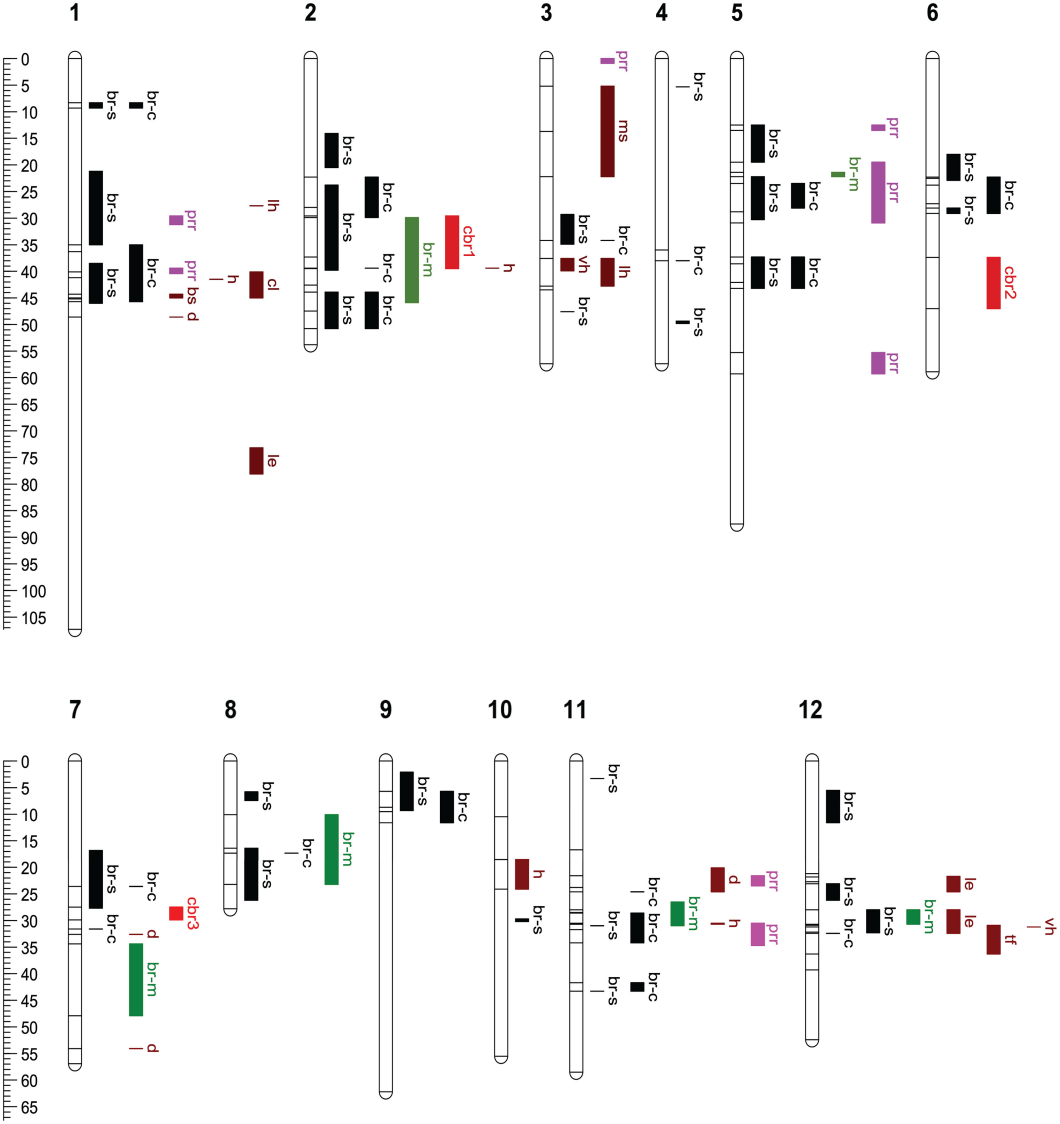
Comparison of QTL intervals for blight resistance, *Phytophthora* root rot resistance and nine morpho-phenological traits. All QTL intervals were drawn according to their positions on the reference genetic map embedded in *C. mollissima* (Vanuxem) reference genome v4.3. The positions of SNPs that delimit QTL intervals are shown as horizontal ticks. Abbreviations of chestnut blight resistance QTLs: br-s, QTLs detected by GWAS using fungal strain specific canker size data; br-c, QTLs by GWAS using combined canker size data; br-m, QTLs by MQM in present study; cbr1 - 3, by MQM in Kubisiak et al. (2013). Abbreviations of other traits’ QTLs: prr, *Phytophthora* root rot (PRR) resistance; lh, for leaf hair; le, leaf emergence; ms, male sterility; vh, vein hair; bs, bud shape; tf, tree form; cl, central leader; h, height; d, diameter. The positions of PRR QTLs were remapped from the publication of Zhebentyayeva et al. (2019). Color scheme for different QTLs: black for BR QTLs by GWAS; green for br-m; red for cbr1-3; pink for prr; brown for nine morpho-phenological traits’ QTLs.

To facilitate the comparison of mapping methods, we mapped SNPs in MQM-detected QTL intervals to the *C. mollissima* reference genome v4.3 and displayed the QTL intervals detected by both methods on **Figure 7**.

MQM in the core subset of F2 family detected six QTLs on six chromosomes. GWAS using combined strain data detected three QTLs (on Chromosome 2, 8, and 11) that had overlapping intervals with MQM detected QTLs. GWAS using fungal strain-specific data detected an additional QTL (on Chromosome 12) having an overlapping interval with an MQM-detected QTL. In total, GWAS (both combined and strain specific data analyses) detected 29 composite QTL intervals distributed across 12 chromosomes, indicating its greater sensitivity, flexibility and resolution than MQM (**Supplementary Tables 4, 5**, **Figure 7**). In comparison to the previously reported three blight resistance QTLs (Cbr1, Cbr2, Cbr3) (Kubisiak et al., 2013), GWAS and MQM in this study confirmed Cbr1 (with overlapping QTL intervals) on chromosome 2 and Cbr3 (with QTLs nearby) on chromosome 7, but not Cbr2 on chromosome 6 (**Figure 7**).

### Comparison of QTL intervals for blight resistance and morpho-phenological traits

Since the central goal of the backcross breeding strategy is to introgress the Chinese chestnut resistance trait into American chestnut, while maintaining the overall “American” phenotype, we were interested in determining if there was any significant overlap between resistance-associated QTLs and genomic intervals controlling archetypal American chestnut traits. For this purpose, we analyzed data accumulated on these families for nine M/P traits that are known to differentiate the two species, including tree growth and form related traits (tree height, stem diameter, tree form, and central leader); leaf and bud morphology traits (leaf hair, vein hair, and bud shape); a phenology trait (leaf emergence); and a reproductive trait (male sterility). A large number (15 out of 19) of the M/P trait QTLs overlap or are close to blight resistance QTLs. However, 12 relatively large effect M/P QTLs concentrate in four regions on chromosomes 1, 3, and 12; while the blight resistance QTLs are more evenly distributed onto 12 chromosomes. For chromosomes 4, 5, 6, 8, and 9, we found blight resistance QTLs, but no M/P QTLs (**Figure 7**).

### Comparison of QTL intervals for blight resistance and *Phytophthora* root rot resistance

To determine if blight and PRR resistance exhibit common or overlapping QTLs, we mapped SNPs in previously published QTL intervals for PRR resistance (Santos et al., 2017; Zhebentyayeva et al., 2019) to the *C. mollissima* reference genome v4.3 and displayed those QTLs in **Figure 7**. The previously identified PRR resistance QTLs were mapped to eight separate regions on chromosome 1, 3, 5, and 11. Three of these (QTLs on chromosomes 1, 5, and 11) overlap with QTLs detected by GWAS using combined strain data. Two additional PRR QTLs (on Chromosome 1 and 5) overlap with QTLs detected by GWAS using strain-specific data (**Figure 7**).

### Identification of candidate genes and tightly linked markers for blight resistance

Investigation of CGs for blight resistance was mainly focused on the 18 QTLs associated with blight resistance detected by combined strain analyses, as the majority of these intervals were also detected by strain-specific analyses. In addition, we incorporated three non-overlapping MQM-detected QTLs into CG analysis because of their strong QTL effects (**Table 1**, **Supplementary Table 4**).

Coupling the genetic mapping results (GWAS and MQM) and reanalyzed transcriptome data obtained from canker and healthy stem samples of American and Chinese chestnuts (Barakat et al., 2009, 2012), we identified 766 CGs that were differentially expressed in American and Chinese chestnut canker tissues and located within the blight resistance QTL intervals. In addition, we assigned 610 Arabidopsis genes homologous to these chestnut genes (**Supplementary Table 8A**).

Considering genes that are tightly linked to the most significant SNP/SSRs detected by GWAS and MQM, we identified a list of 129 high-priority CGs for blight resistance across these 21 QTLs (**Supplementary Table 8B**). In addition to CGs, we identified 67 highly significant markers in the GWAS (p < 0.005) or MQM (1 LOD interval) detected QTLs. Preliminary analysis found that most of these markers showed appreciable allelic diversity between American and Chinese chestnuts, implying their potential usefulness in MAS for blight resistance (**Supplementary Table 9**).

### Identification of candidate genes for morpho-phenological traits

We performed CG searches for four M/P traits: leaf hair, vein hair, male sterility, and leaf emergence. All four M/P traits exhibited a highly significant QTL peak (p < 2.2 × 10^-5^, Bonferroni corrected p value cutoff) in Manhattan plots (**Figure 6**). CGs were only mined from these high confidence and high resolution QTLs as detailed in **Supplementary Data 5**.

Leaf hair and vein hair share a highly significant QTL peak on chromosome 3 (**Figure 6**), suggesting that they are potentially regulated by the same set of genes. In this QTL (10 Mb region, **Supplementary Table 10**), we identified two MIXTA-MYB type genes, Caden.03G223800 and Caden.03G224000, that appear to be the most likely CGs controlling leaf/vein hair in chestnut, as these genes are putative orthologs of poplar genes responsible for trichome formation (see dendrogram in **Supplementary Figure 3**) (Bewg et al., 2022). In land plants, MIXTA-MYB transcription factors frequently induce epidermal cell differentiation (Xu et al., 2021).

For male sterility, we identified a QTL (7.84 Mb region, **Supplementary Table 10**) on chromosome 3 containing 31 pentatricopeptide repeat (PPR) protein-coding genes (**Supplementary Table 11**). These appear to be CGs controlling male sterility, as they are known to be restorers of male fertility in rice (Toriyama, 2021) and other plant species having cytoplasmic male sterility/fertility restorer systems (Chen and Liu, 2014). Since there is evidence of cytoplasmic male sterility in TACF chestnut breeding materials (Shi and Hebard, 1997; Sisco, 2004), it is reasonable to hypothesize that we have potentially mapped nuclear restorer genes segregating in some of our crosses that may contain male sterile cytoplasmic/nuclear gene interactions. Further studies are needed to test this hypothesis.

For leaf emergence, we identified a QTL (27.5 Mb region, **Supplementary Table 10**) on chromosome 12. Within this region, we identified 30 CGs including three putative orthologs of Polycomb repressive complex 2 subunits *VIN3* (Caden.12G091300), *EMF2* (Caden.12G128000), *FRIGIDA-LIKE 3* (Caden.12G191300), and three MADS-box transcriptional factors (Caden.12G090000, Caden.12G141600, Caden.12G176700) involved in vegetative growth and flowering in *Arabidopsis*. The other CGs include growth-regulating and stress-responsive transcription factors, hormonal regulation networks, histone modification and chromosome remodeling factors (**Supplementary Table 12**). Notably, a portion of this QTL (from gene Caden.12G089000 to Caden.12G095400 in *C. dentata* genome v1.1) shows conserved synteny with the sequence harboring a major QTL on G1 for chill requirement and bloom date in peach (Fan et al., 2010).

## Discussion

### GWAS with inter-related complex families

For this research, we utilized data collected on >1100 trees developed and evaluated as part of TACF’s ongoing blight resistance breeding program. In addition to blight resistance (evaluated as canker size), several M/P traits, which taxonomically distinguish the parental species (*C. dentata* and *C. mollissima*), were measured or scored. As the phenotypic data accumulated over time and marker technologies progressed (from RFLP, RAPD, and SSR to SNP), subsets of these trees were genotyped. Earlier publications reported on these trees and marker datasets (e.g., Kubisiak et al., 1997, 2013), focusing on mapping blight resistance. In these earlier studies, the tree dataset was confined to one inter-species hybrid F2 family and either RAPD and RFLP markers or SSR and SNP markers. In the current study, we genotyped the entire set of trees (the expanded F2 and several backcross families) with a moderate-density SNP array, combined these data with the available SSR marker and accumulated phenotypic data, and analyzed the marker-trait data using both QTL mapping and GWAS methods. Our primary interests were to improve the understanding of the genetic architecture of blight resistance by including additional sources of resistance and larger progeny sample sizes, as well as a higher marker density than previously available. In addition, we wanted to determine the genetic relationships between blight resistance and traits that distinguish the parental species, as both issues are critical to the successful implementation of inter-species backcross breeding programs. Secondarily, we were interested in comparing results between QTL mapping and GWAS methods and between the F2 and various backcross families.

Initially we utilized a single-family linkage mapping method, namely, MQM and found it only suitable for analysis of a core part (i.e., cross made in one direction with outcrosses removed) of the MahF2 family, and not in families with more complex pedigrees. QTL mapping requires a sufficiently large (100s or more trees) family derived from the same cross (full-sib family) with genotyped parents to ensure that QTLs with reasonable resolution can be detected with high statistical confidence. The complexities in the current study include multiple, outbred (heterozygous and heterogenous) donor and recipient parent trees, resulting in several bi-parental crosses with small family sizes, and some progeny that were not of the intended pollination (i.e., outcrosses). For example, the number of trees tested from each cross in the MahB1 family ranges from 1 to 17 (**Figure 1B**); while 690 trees from 24 crosses in the GraB3 family (involving 12 B2 males and 20 American chestnut females) were evaluated, about 29 trees per cross (**Figure 1E**). Only the ClaB2 family contained progeny trees from the same bi-parental cross, although the family size (n = 76) is still relatively small for optimal QTL mapping (**Figure 1D**).

Given the complexity of the available pedigrees, we looked into the literature for analytical approaches beyond single-family QTL methods. One approach, a joint multi-family linkage analysis (Hayashi and Iwata, 2009; Ogut et al., 2015), requires lines of recombinant inbreds or doubled haploids derived from crosses with a common reference line. A second approach uses a Bayesian framework to analyze multiple families, but each family requires a known pedigree and moderately large family sizes (Bink et al., 2014; Rawandoozi et al., 2023). Neither of these approaches fit the complexity and imbalance of our experimental materials, thus we considered GWAS across these families with the possibility of directly comparing GWAS to single-family results obtained with MQM. Traditionally, GWAS in crop plants uses a population of genetically diverse and unrelated lines, either inbred or clonally propagated to improve the accuracy of phenotypic assessments. Here we applied GWAS to multiple complex families, necessarily involving related, outbred trees without clonal replication. Specifically, we utilized GWAS analyses at multi- and single-family levels, recognizing that the relatedness among trees within families results in large linkage disequilibrium blocks affecting the resolution of mapping. However, the primary objective of this study was to detect and delineate QTLs or gene regions, instead of identifying CGs per se or highly significant quantitative trait nucleotides. Nonetheless, transcriptome analysis and comparative mapping of the detected QTLs did highlight specific genes (**Supplementary Data 5**, **Supplementary Tables 8, 10-12**) for disease resistance and M/P traits that had been identified in other species, validating this approach as a first step in characterizing the genetic architecture of blight resistance and identifying CGs for downstream applications.

### Normalization of phenotypic data

TACF’s breeding program is a decades-running cooperative effort with the seedlings utilized in this research being planted and tested at two different locations (Virginia and Pennsylvania) and in various years (as early as 1991 and as late as 2011). The environmental factors including soil type, rainfall amount and pattern, and seasonal air temperatures undoubtedly affected tree vigor, canker growth, and the M/P traits. Additionally, as described above, there were some differences in canker size scoring methods, in that some cankers were measured in length and width, and others in length only.

To minimize the influences of resistance sources, environmental factors, and scoring methods, we normalized the canker size data from each cohort (family × inoculation year-location) using z-scores. This allowed us to combine data across cohorts for the multi-family GWAS.

Prior to normalization, we noticed differences of genetic diversity in the PCA between and within families. The F2 family showed greater genetic diversity as expected than the backcross families (**Figure 2**). If blight resistance is determined by many small effect genes, then later backcross generations would inevitably lose some resistance and have a larger mean (less resistance) and smaller standard deviation of canker size. Normalization by z-scores and combining all seven cohorts into one dataset would likely dampen the F2 family’s impact in the pooled population. For this reason, we performed separate GWAS analysis for the F2 family and the pool of four backcross families for blight resistance.

### Genetic control of blight resistance

Recognizing the difficulty of accurate quantification of blight resistance and data integration over cohorts, we collected multiple lines of evidence to complement and cross validate our results. We made comparisons of multi-family vs. single-family GWAS, multi-family GWAS vs. single-family F2 MQM, and present vs. past mapping studies. Combining the findings from these analyses and comparisons, we identified and delineated 31 QTLs across the chestnut genome (involving all 12 chromosomes), most of them with small to moderate effects on blight resistance as measured by canker size (**Table 1**, **Supplementary Tables 4, 5**). In this research, two fungal strains with different virulence levels were used to evaluate the resistance levels of each inoculated tree. We detected the existence of host and fungal strain interactions, in that some QTLs were detected for only one strain, while others were detected for both strains (**Table 1**, **Supplementary Table 5**). Although the two pathogen strains used here represent moderately to highly virulent strains, they do not constitute an inclusive collection, suggesting that future work with additional strains may be necessary to find common QTLs broadly associated with blight resistance. Trees with “good” alleles at such loci would likely provide long lasting protection against evolving strains of the pathogen (Nelson et al., 2018). It might be easiest to first detect highly virulent strains by sampling severe cankers on previously resistant breeding lines. Likewise, isolations could be made from cankers with reduced severity.

Coupling transcriptomics with GWAS and QTL mapping studies provides the possibility of uncovering specific CGs that influence blight resistance. Utilizing existing EST data from previous studies (Carlson, 2008; Barakat et al., 2009, 2012), we identified 129 high-priority CGs (**Supplementary Table 8B**) that are tightly linked (within 0.5cM distance) to the most significant SNP/SSRs (**Supplementary Table 9**). Within this collection of CGs, we noted several sets of genes that are related to various defense responses. One set of genes involve signaling activities across cellular membranes of either the plasmalemma, vacuolar or multi-vesicular body systems. Another set contains ribosomal protein related genes, including the *EIF3* gene (Cm_g17246) as well as protein-protein interacting genes such as the TRAF-like domain gene (Cm_g10451). These genes are known to be associated with plant resistance responses and play a central role in signaling stress responses in plants (Qi et al., 2022; Son and Park, 2023). In addition, we note that several CGs (Cm_g5365, Cm_g10199, Cm_g4882, Cm_g8337, Cm_g10195, Cm_g33967, and Cm_g819) encode predicted proteins with actions like those identified in lesion mimic mutant (LMM) in Liu et al. (2022). LMMs spontaneously induce cell death phenotypes that can activate the overall immune response, enhancing disease resistance (Wu et al., 2008).

When surveying the list of high-priority CG/DEGs in **Supplementary Table 8B**, two key differences are evident between genes up-regulated in American or Chinese chestnut cankers. First, the genes up-regulated in American chestnut cankers (66%) outnumber those up-regulated in Chinese chestnut cankers (34%). This suggests that the host cellular actions in these two species are moving in different directions or have not achieved the same stage when the canker tissues were sampled (Barakat et al., 2009, 2012). One hypothesis for this is that the Chinese chestnut host, having co-evolved with *C. parasitica*, recognizes the pathogen and quickly mounts a defense response, while the American chestnut host does not recognize the pathogen early enough and thus lags in its defense response, giving the pathogen an advantage in canker progression. Very early defense response (within nine hours after inoculation) by Chinese chestnut was noted recently by Nie et al. (2023). They reported that down-regulated genes outnumber up-regulated genes at nine hours, which appears to be a critical timepoint in the infection process. This down-regulation in Chinese chestnut cankers was also evident in our transcriptome/QTL analyses, suggesting that the host cell machinery is focusing on specific defense pathways at the expense of homeostatic growth and development pathways. The second key difference is that up-regulated American chestnut genes are over-represented in protein metabolism (i.e., translation control, ribosomal structure, protein degradation), while Chinese chestnut cankers have bias for up-regulation of proteins involved in transcription and RNA processing, again reflective of potentially different stages or focus of defense response systems. Unfortunately, Nie et al. (2023) did not study American chestnut response for comparison.

Interestingly, both our work and that of Nie et al., (2023) noted a *PR1* candidate (Cm_g826 in this study) that is an up-regulated gene in Chinese chestnut, encoding a CAP (Cysteine-rich secretory proteins, Antigen 5, and Pathogenesis-related 1 protein) superfamily protein. This gene is in QTL C3 on chromosome 2 (**Supplementary Table 4**) corresponding to part of the previously mapped Cbr1 QTL interval in Kubisiak et al. (1997, 2013). PR1 proteins have been shown in previous studies to play a substantial role in plant resistance to a few pathogens and operate in the apoplast where initial contacts occur between host and pathogen (for review, see Han et al., 2023). Additionally, PR1 proteins bind lipids and are implicated in lipid signaling and defense responses. Lipid metabolism genes were the second most abundant class in our blight QTL intervals (**Supplementary Table 8**). Finally, QTL C3 was noted in different crosses using different Chinese chestnut resistance sources (**Supplementary Table 4**), thus *PR1* should be a high-priority gene for further study.

### Breeding for disease resistant, phenotypically “American” chestnut trees

For backcross breeding to be successful, the targeted blight resistance trait must be transferred to the recipient species (American chestnut) with as few other donor alleles (traits) as possible. The more complex the target trait, the greater the chance that linkage drag will bring undesirable donor traits along with the desired trait (i.e., blight resistance). With 31 blight resistance QTLs identified on 12 chromosomes, this presents a significant challenge for a clean transfer of blight resistance of Chinese chestnut to American chestnut. In addition, the chance of obtaining backcross hybrid trees with a resistance allele at every QTL is extremely low. However, within families, often only two to three QTLs were detected. This may be due to the small family sizes or may reflect a more complex genetic structure of blight resistance than a collection of separate, non-interacting alleles each conferring partial resistance.

To complicate matters further, we found most QTLs for M/P traits to overlap or be positioned quite close to those for blight resistance. Having both blight resistance and M/P traits mapped enables marker-informed selection for genetic recombinations that combine resistance and American chestnut M/P characteristics. Moreover, we note that blight resistance QTLs tend to distribute across the whole genome, while the M/P trait QTLs are concentrated on chromosomes 1, 3, and 12 (**Figure 7**). The different distribution patterns of blight resistance and M/P QTLs at least partly explain the lack of correlations between canker size and the M/P traits (**Figure 4**, **Supplementary Table 3**), and suggest that selection for resistant marker alleles could focus on chromosomes 2, 4 to 11, leaving chromosomes 1, 3 and 12 to be affected by selection for American chestnut traits.

PRR is another disease that severely limits the establishment of American chestnut and has implications for restoration, especially in the southern portion of the species’ range. To evaluate the potential for selecting both chestnut blight and PRR resistances, we compared the genomic locations of the blight resistance QTLs with the recently reported PRR QTLs in a *C. sativa* × *C. crenata* cross (Santos et al., 2017) and in multiple *C. dentata* × *C. mollissima* crosses (Zhebentyayeva et al., 2019). Mapping both the blight resistance and PRR QTLs to the *C. mollissima* reference genome v4.3, showed that five PRR QTLs indeed overlapped with blight resistance QTLs on chromosome 1, 5, and 11, including the two strongest PRR QTLs on chromosomes 5 and 11 in both previous PRR mapping studies (**Figure 7**). However, preliminary analysis of DEGs from the *P. cinnamomi* studies do not show correlations with blight resistance CGs in these overlapping areas (T. Zhebentyayeva, personal communication). As both the blight fungus and *P. cinnamomi* are necrotrophic in all or part of their life cycle, respectively, this result suggests that resistance in chestnut may target different aspects of each pathogen’s biology and the QTL overlaps are not due to common genes in a general necrotrophic response. Validating any of these in chestnut remains a challenge, but progress in chestnut somatic embryogenesis and gene transformation (Kong et al., 2014; Newhouse et al., 2014), including genome editing, provide the required technologies for such work. Whether or not a common underlying resistance pathway exists in blight and PRR disease, more molecular marker research should be conducted to identify QTL haplotypes that positively contribute to both blight and PRR disease resistance in the overlapping QTL regions.

With an improved understanding of the genetic architectures of blight and PRR resistance in chestnut, we can consider approaches of MAS. One approach is to partition the chestnut genome into three components (blight resistance, PRR resistance, and the remainder) followed by characterizing the three components for their relative Chinese and American chestnut marker/gene allelic content. The results reported here suggest that the blight resistance component can be marked by 67 SNPs across 21 important QTLs. Similarly, informative markers could be identified and then characterized for the PRR resistance QTLs. In this approach, MAS would be a matter of genotyping the three sets of markers to determine each set’s Chinese or American chestnut allelic content among the genotyped candidate trees, and then selecting trees with high Chinese chestnut allelic content (indicating high relative resistance) for the blight and PRR resistance components and high American chestnut allelic content for the remainder (e.g., M/P traits) component.

An alternative approach is to apply genomic selection. Application of genomic selection is being investigated in breeding programs of woody perennial species including chestnut (Westbrook et al., 2020), fruit trees and bushes (Roth et al., 2020; Ferrão et al., 2021; Kostick et al., 2023; Sun et al., 2024), and other tropical fruit and plantation tree species (Seyum et al., 2022). It is evident that efficient genomic selection needs a relatively large training population using comparable criteria for phenotyping and having a similar genetic background to the trees under selection. This may be difficult to implement across a multi-state breeding network with diverse germplasm that is required for TACF’s species restoration program. In contrast, the MAS approach outlined above, requires only knowing where the resistance regions of the genome are located and that the Chinese chestnut marker or gene allelic content of these regions can be distinguished from their American chestnut allelic content. In this regard, as outlined in Ferrão et al. (2021), breeding schemes that utilized MAS early in the process for large effect QTLs followed by genomic selection in later generations to incorporate smaller effect genomic regions showed promise for streamlining the breeding for the trait of interest. In time, as additional resistance regions are identified (e.g., through mapping additional Chinese chestnut ancestral trees, by discovering additional resistance mechanisms) or these regions are refined and validated or even refuted, the delineation of the three components can be adjusted to improve MAS with limited disruption in the selection and breeding activities.

## Data availability statement

The data presented in the study are deposited in the TreeGenes repository at https://treegenesdb.org/tpps/details/TGDR1224, accession number TGDR1224.

## Author contributions

SF: Formal analysis, Investigation, Methodology, Validation, Visualization, Writing – original draft, Writing – review & editing. LLG: Formal analysis, Investigation, Methodology, Validation, Visualization, Writing – original draft, Writing – review & editing. FVH: Conceptualization, Data curation, Investigation, Methodology, Resources, Validation, Writing – original draft, Writing – review & editing. TZ: Formal analysis, Investigation, Methodology, Validation, Visualization, Writing – original draft, Writing – review & editing. JY: Formal analysis, Methodology, Visualization, Writing – original draft, Writing – review & editing. PHS: Resources, Validation, Writing – review & editing. SFF: Investigation, Resources, Validation, Writing – review & editing. MES: Formal analysis, Methodology, Validation, Writing – review & editing. AGA: Formal analysis, Supervision, Validation, Writing – original draft, Writing – review & editing. CDN: Conceptualization, Formal analysis, Funding acquisition, Resources, Supervision, Writing – original draft, Writing – review & editing.
