# Supplementary material for: Mapping QTLs for blight resistance and morpho-phenological traits in inter-species hybrid families of chestnut (Castanea spp.)
Source: Front Plant Sci. 2024 Apr 8;15:1365951. doi: 10.3389/fpls.2024.1365951 (PMC11033410; doi:10.3389/fpls.2024.1365951)
Supplement: Supplementary file 1 [file DataSheet_1.zip › Data Sheet 1/List of supplementary materials.docx]

**List of supplementary materials**

**Supplementary Tables:**

**Supplementary Table 1.** Statistics for GWAS analysis for blight resistance and morpho-phenological traits

**Supplementary Table 2.** Planting and phenotyping times of mapping families

**Supplementary Table 3.** Pairwise correlation coefficients between four dimensions of canker size and nine mopho-phenological traits

**Supplementary Table 4.** QTL intervals for blight resistance in chestnut detected by GWAS (with combined canker size data) and MQM.

**Supplementary Table 5.** QTL intervals for blight resistance in chestnut detected by GWAS (with SG2-3 or Ep155 induced canker size data) and MQM.

**Supplementary Table 6.** The inter-species F2 genetic map for MQM with the expanded MahF2 family

**Supplementary Table 7.** QTL intervals for nine morpho-phenological traits in chestnut detected by GWAS

**Supplementary Table 8A.** The master list of candidate genes for blight resistance

**Supplementary Table 8B.** The list of high-priority candidate genes for blight resistance

**Supplementary Table 9.** The list of SNP markers for potential marker-assisted selection and their allele frequencies in 3 Chinese vs. 13 American chestnut trees.

**Supplementary Table 10.** Markers used for mining C. dentata v1.0 genome for candidate genes underlying leaf and vein hair, male sterility, and leaf emergence.

**Supplementary Table 11.** List of PPR genes within genomic region associated with male sterility.

**Supplementary Table 12.** List of candidate genes for leaf emergence.

**Supplementary Data:**

**Supplementary Data 1.** Phenotypic data of 1124 chestnut interspecies hybrid trees

**Supplementary Data 2.** Genotypic data of 3920 SNPs of 1131 chestnut interspecies hybrid trees.

**Supplementary Data 3.** EST or genomic sequences that harbor 3959 SNP/SSR markers

**Supplementary Data 4.** The updated reference genetic map for Chinese chestnut.

**Supplementary Data 5.** Identification of candidate genes for morpho-phenological traits.

**Supplementary Figures:**

**Supplementary Figure 1.** The alternative pedigree of Graves backcross family

**Supplementary Figure 2.** Manhattan plots showing significant SNP/QTLs detected by GWAS analysis of blight resistance in chestnut using strain specific or combined canker size data.

**Supplementary Figure 3.** Dendrogram based on alignment of proteins sequences of the MIXTA-type R2R3 MYB transcriptional factors involved in trichome formation in Arabidopsis thaliana (AT5G15310, AT3G01140), tomato (Solyc02g088190), peach (Prupe.5G196100) and poplar (MYB186 -Potri.008G089200, MYB138 - Potri.008G089700, MYB38 - Potri.010G165700).
