# Supplementary material for: Mapping QTLs for blight resistance and morpho-phenological traits in inter-species hybrid families of chestnut (Castanea spp.)
Source: Front Plant Sci. 2024 Apr 8;15:1365951. doi: 10.3389/fpls.2024.1365951 (PMC11033410; doi:10.3389/fpls.2024.1365951)
Supplement: Supplementary file 1 [file DataSheet_1.zip › Data Sheet 1/Supplementary Data 5.docx]

**Supplementary Data 5. Identification of candidate genes for morpho-phenological traits.**

**Leaf and vein hair**

Due to overlapping QTL regions for leaf and vein hair, we consider two traits as related and controlled by the same genes. Thirty-one SNPs associated with traits spread over 35.74 Mb-region (chr03:21637816-57391887 in the Ellis genome) including 1,455 protein-coding genes. Using more stringent criteria (*p* < 1E-05 for SNPs associated with traits), we narrowed down the candidate gene search to a 10 Mb-region with 18 SNPs covering 526 predicted genes (Supplementary Table 10). In land plants, epidermal cell differentiation is frequently induced by MYB-MIXTA-type transcriptional factors (Xu et al., 2021). Presumably, leaf/vein hair in chestnut could be under similar genetic control. Altogether, seven MYB genes were annotated within genomic region of interest. In proximity of the most significant marker CCallv2c3338_107 (~ 1Mb apart, p < 2.26E-21), we identified two genes Caden.03G223800 and Caden.03G224000, putative orthologs of poplar MYB-genes responsible for trichome formation in poplar leaves (Bewg et al., 2022; Han et al., 2022). An UPGMA dendrogram of protein alignment of Caden.03G223800 and Caden.03G224000 and MIXTA-type MYB proteins from poplar (Potri.008G089200, Potri.008G089700 and Potri.010G165700) and other plants is shown on Figure 1.


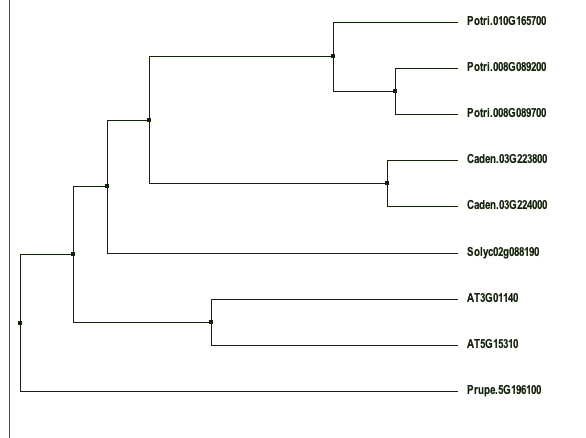


Figure 1. Dendrogram based on alignment of proteins sequences of the MIXTA-type R2R3 MYB transcriptional factors involved in trichome formation in Arabidopsis thaliana (AT5G15310, AT3G01140), tomato (Solyc02g088190), peach (Prupe.5G196100) and poplar (MYB186 -Potri.008G089200, MYB138 - Potri.008G089700, MYB38 - Potri.010G165700).

**Male sterility**

Cytoplasmic male sterility (CMS) is a maternally inherited trait caused by disrupted interaction between nuclear and organellar genomes. Nucleus-encoded pentatricopeptide repeat (PPR) proteins essential for biogenesis of organellar RNA are the most likely candidates for the control of CMS (Toriyama, 2021). Genomic region of 7.84 Mb (chr03: 432996 - 8277450 in American chestnut Ellis genome) was delineated with 11 SNPs associated with male sterility and covered 416 protein-coding regions (Supplementary Table 10). Of these, 31 genes were annotated as PPR-encoded proteins (Supplementary Table 11). Four of them (Caden.03G014000, Caden.03G017400, Caden.03G019200 and Caden.03G027500) have signature domains (E+ and DYW on Figure 2) required for site-specific editing of organellar RNAs by nucleic acid deaminases (Wagoner et al., 2015). Also, remaining PPR proteins are structurally similar with nucleus-encoded fertility restorers AT1G12700 and LOC_Os10g30760 in *A. thaliana* (Hölzle et al., 2011) and rice (Wang et al., 2006).


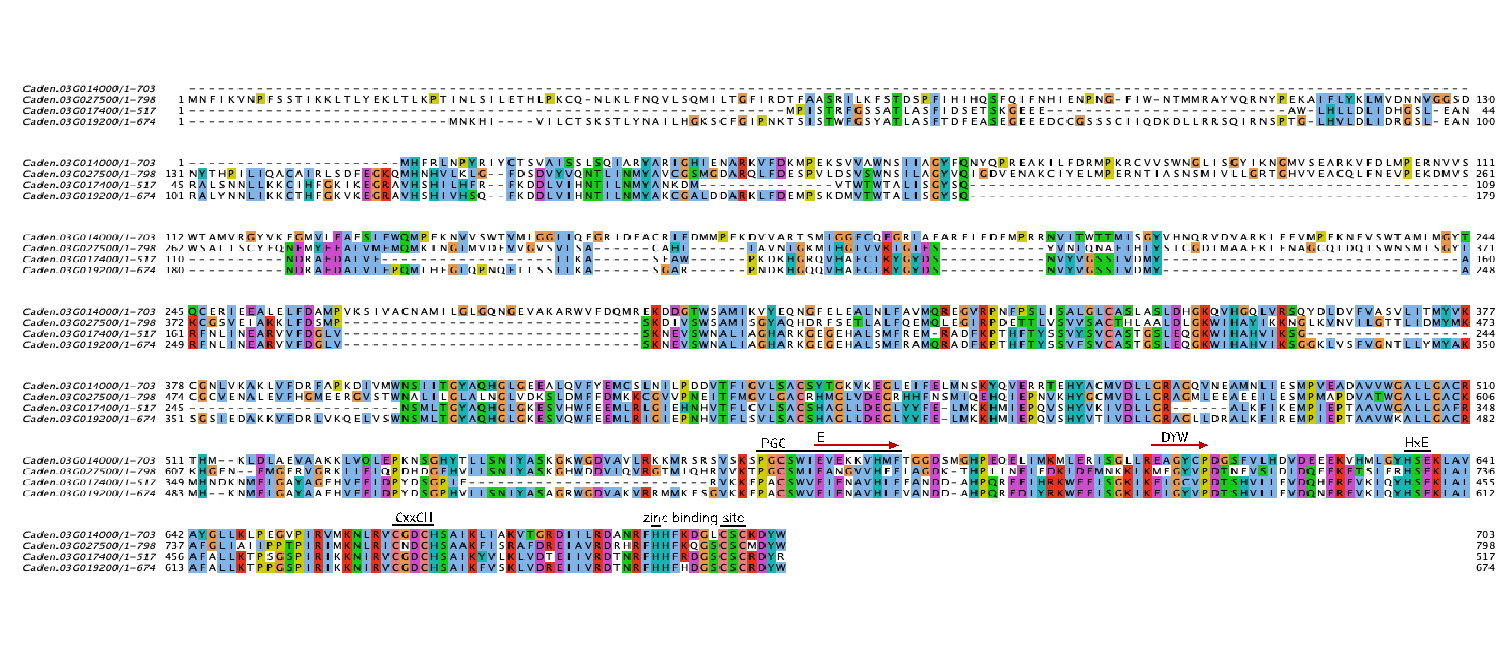


Figure 2. Protein alignment of putative PPR-DYW deaminases Caden.03G014000, Caden.03G017400, Caden.03G019200 and Caden.03G027500 identified in the region associated with male sterility in chestnut.

**Leaf emergence**

For temperate forest trees, leaf emergence is an important adaptive trait controlled by cumulative hours at growth-favoring conditions after winter dormancy. In total, 19 SNP markers associated with this trait spread over genomic region of 27.5 Mb from Caden.12G08900 to Caden.12G201300 in the Ellis genome, while the eight most significantly associated markers (*p* < 1E-09) cover a smaller region of 1.1 Mb between Caden.12G108000 and Caden.12G111400 (Supplementary Table 10). Notably, a genomic region of 2.1 Mb from Caden.12G089000 to Caden.12G095400 was syntenic to sequence underlying major QTL interval on G1 for chill requirement and bloom date in peach (Fan et al., 2010). Recently, highly confident allele-specific PCR test for this region was developed to predict chill requirements in peach germplasm and breeding materials (Demirel et al., 2023). Altogether, we inspected 941 annotated genes to identify homologs of key regulators of vernalization, growth and climatic adaptation in *Arabidopsis* (Preston and Fjellheim, 2022) as well as elements of cold-responsive molecular network in perennial trees (Abbott et al., 2015). A list of 30 candidate genes for control of leaf emergence is presented in Supplementary Table 12. The most abundant group of putative candidate genes was composed of nine PETALA2/ETHYLENE RESPONSE FACTORs (*AP2*/*ERF*), mediators of stress responses and developmental programs (Mizoi et al., 2012): one ETHYLENE-RESPONSIVE ELEMENT BINDING PROTEIN (*EREB*), two DEHYDRATION-RESPONSIVE ELEMENT-BINDING PROTEIN (*DREB*) and six ETHYLENE-RESPONSIVE TRANSCRIPTION FACTORs (*ERF*). Two genes, Caden.12G091300 and Caden.12G128000, were putative orthologs of the VERNALIZATION-INSENSITIVE PROTEIN 3 (*VIN3*) and the *EMBRYONIC FLOWER2* (*EMF2*), subunits of the Polycomb Repressive Complex 2 (*PRC2*) involved in vernalization response in *Arabidopsis*, respectively. Ten genes were annotated as functional enzymes involved in post-translational modification of histones (methylation and acetylation) and DNA-targeting chromatin remodeling. A group of MADS-box transcriptional factors included Caden.12G090000, an ortholog of the *DAM* genes associated with chill requirement in *Prunus*; floral homeotic MADS-box protein Caden.12G14160, a putative homolog of *PISTILLATA* (AT5G20240) required for specification of petal and stamen identities in *Arabidopsis*; and Caden.12G176700, a homolog of *AGAMOUS-LIKE 82* gene AT5G55690. The list of candidate genes also included six growth regulating transcriptional factors and hormonal regulation network genes (Supplementary Table 12).

**Acknowledgement**

*C. dentata* v1.1 genome assembly was produced by the HudsonAlpha Genome Sequencing Center and The American Chestnut Foundation and made available by the Department of Energy's Joint Genome Institute.

**References**

Abbott, A. G., Zhebentyayeva, T., Barakat, A., and Liu, Z. (2015). The Genetic Control of Bud-Break in Trees. *Adv Bot Res* 74, 201–228. doi: 10.1016/bs.abr.2015.04.002.

Bewg, W. P., Harding, S. A., Engle, N. L., Vaidya, B. N., Zhou, R., Reeves, J., et al. (2022). Multiplex knockout of trichome-regulating MYB duplicates in hybrid poplar using a single gRNA. *Plant Physiol* 189, 516–526. doi: 10.1093/plphys/kiac128.

Demirel, G., Calle, A., Lawton, J. M., Atagul, O., Fu, W., and Gasic, K. (2023). Ppe.CR.1 DNA test for predicting chilling requirement in peach. *Sci Rep* 13. doi: 10.1038/s41598-023-27475-w

Fan, S., Bielenberg, D. G., Zhebentyayeva, T. N., Reighard, G. L., Okie, W. R., Holland, D., et al. (2010). Mapping quantitative trait loci associated with chilling requirement, heat requirement and bloom date in peach (Prunus persica). *New Phytologist* 185, 917–930. doi: 10.1111/j.1469-8137.2009.03119.x.

Han, G., Li, Y., Yang, Z., Wang, C., Zhang, Y., and Wang, B. (2022). Molecular Mechanisms of Plant Trichome Development. *Front Plant Sci* 13. doi: 10.3389/fpls.2022.910228.

Hölzle, A., Jonietz, C., Törjek, O., Altmann, T., Binder, S., and Forner, J. (2011). A Restorer of Fertility-like PPR gene is required for 5a′-end processing of the nad4 mRNA in mitochondria of arabidopsis thaliana. *Plant Journal* 65, 737–744. doi: 10.1111/j.1365-313X.2010.04460.x.

Mizoi, J., Shinozaki, K., and Yamaguchi-Shinozaki, K. (2012). AP2/ERF family transcription factors in plant abiotic stress responses. *Biochim Biophys Acta Gene Regul Mech* 1819, 86–96. doi: 10.1016/j.bbagrm.2011.08.004.

Preston, J. C., and Fjellheim, S. (2022). Flowering time runs hot and cold. *Plant Physiol* 190, 5–18. doi: 10.1093/plphys/kiac111.

Toriyama, K. (2021). Molecular basis of cytoplasmic male sterility and fertility restoration in rice. *Plant Biotechnology* 38, 285–295. doi: 10.5511/plantbiotechnology.21.0607a.

Wagoner, J. A., Sun, T., Lin, L., and Hanson, M. R. (2015). Cytidine deaminase motifs within the DYW domain of two pentatricopeptide repeat-containing proteins are required for site-specific chloroplast RNA editing. *Journal of Biological Chemistry* 290, 2957–2968. doi: 10.1074/jbc.M114.622084.

Wang, Z., Zou, Y., Li, X., Zhang, Q., Chen, L., Wu, H., et al. (2006). Cytoplasmic male sterility of rice with Boro II cytoplasm is caused by a cytotoxic peptide and is restored by two related PPR motif genes via distinct modes of mRNA silencing. *Plant Cell* 18, 676–687. doi: 10.1105/tpc.105.038240.

Xu, B., Taylor, L., Pucker, B., Feng, T., Glover, B. J., and Brockington, S. F. (2021). The land plant-specific MIXTA-MYB lineage is implicated in the early evolution of the plant cuticle and the colonization of land. *New Phytologist* 229, 2324–2338. doi: 10.1111/nph.16997.
