# Supplementary material for: Mapping QTLs for blight resistance and morpho-phenological traits in inter-species hybrid families of chestnut (Castanea spp.)
Source: Front Plant Sci. 2024 Apr 8;15:1365951. doi: 10.3389/fpls.2024.1365951 (PMC11033410; doi:10.3389/fpls.2024.1365951)
Supplement: Supplementary file 1 [file DataSheet_1.zip › Data Sheet 1/Supplementary Figure 1 to 3.docx]

**Supplementary Figure 1**. The alternative pedigree of Graves backcross family. The red colored trees are Chinese chestnuts serving as blight resistance donors. The blue colored trees are American chestnut. The Graves family here is a backcross 2 (BC2) family.

**Supplementary Figure 2**. Manhattan plots showing significant SNP/QTLs detected by GWAS analysis of blight resistance in chestnut using strain specific or combined canker size data. BC, all backcross families; MahF2, Mahogany F2 family; MahB1, Mahogany B1 family; NanB1, Nanking B1 family; ClaB2, Clapper B2 family; GraB3, Grave B3 family. The upper two rows show the results from GWAS using canker size data induced by fungal strain SG2-3. The middle two rows show the results from GWAS using canker size data induced by fungal strain Ep155. The bottom two rows show the results from GWAS using combined canker size data. In each plot, each dot represents a SNP. The X-axis shows the positions of SNPs in the Chinese chestnut reference genome v4.3. Numbers "1" to "12" on the X-axis represent 12 chromosomes in the chestnut genome. Number "0" represents the unmapped SNPs. The Y-axis shows the significance level (negative base 10 logarithm of p value) for each SNP tested. The dashed horizontal line represents the genome-wide significance threshold adopted in this study. SNPs above the threshold line are significant and considered for their potential in delineating QTLs.


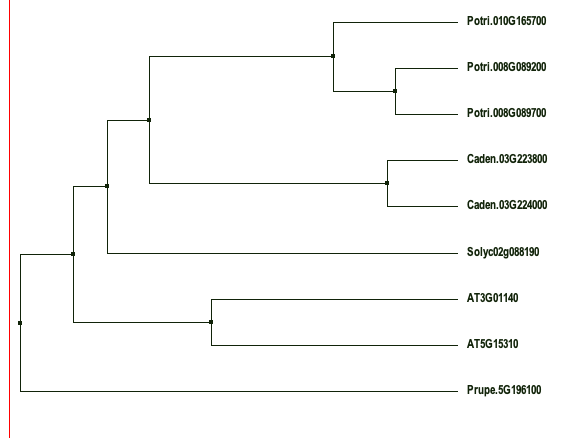


**Supplementary Figure 3**. Dendrogram based on alignment of proteins sequences of the MIXTA-type R2R3 MYB transcriptional factors involved in trichome formation in *Arabidopsis thaliana* (AT5G15310, AT3G01140), tomato (Solyc02g088190), peach (Prupe.5G196100) and poplar (MYB186 -Potri.008G089200, MYB138 - Potri.008G089700, MYB38 - Potri.010G165700).
